# Illumination of the Spatial Order of Intracellular pH by Genetically Encoded pH-Sensitive Sensors

**DOI:** 10.3390/s131216736

**Published:** 2013-12-05

**Authors:** Mojca Benčina

**Affiliations:** 1 Laboratory of Biotechnology, National Institute of Chemistry, 1000 Ljubljana, Slovenia; 2 Center of Excellence EN-FIST, 1000 Ljubljana, Slovenia; E-Mail: mojca.bencina@ki.si; Tel.: +386-1-4760-334; Fax: +386-1-4760-300

**Keywords:** pH homeostasis, intensity-based pH-sensitive sensor, ratiometric transgene pH indicator, flow cytometry, microscopy

## Abstract

Fluorescent proteins have been extensively used for engineering genetically encoded sensors that can monitor levels of ions, enzyme activities, redox potential, and metabolites. Certain fluorescent proteins possess specific pH-dependent spectroscopic features, and thus can be used as indicators of intracellular pH. Moreover, concatenated pH-sensitive proteins with target proteins pin the pH sensors to a definite location within the cell, compartment, or tissue. This study provides an overview of the continually expanding family of pH-sensitive fluorescent proteins that have become essential tools for studies of pH homeostasis and cell physiology. We describe and discuss the design of intensity-based and ratiometric pH sensors, their spectral properties and pH-dependency, as well as their performance. Finally, we illustrate some examples of the applications of pH sensors targeted at different subcellular compartments.

## 1. Introduction

The regulation and homeostasis of pH within a cell and its subcellular compartments are crucial for the viability of any living cell, from the simplest prokaryotes to complex multicellular organisms. Stringent pH requirements are necessary for efficient metabolism, protein stability, ion channel activity, membrane trafficking, protein sorting, and proteolytic processing of proteins. Cellular compartments are protected from rapid, local changes in pH by intrinsic buffering capacity that is provided by various intracellular weak acids and bases. A more dynamic network for controlling intracellular pH (pH_i_) is pH homeostasis, which is composed of an ensemble of well-coordinated ion carriers, such as ion permeable channels, transporters, and pumps [1–4].

An important step forward in the understanding pH homeostasis has been achieved with the development of genetically encoded fluorescent pH-sensitive sensors, which provide a means for spatial and temporal imaging of pH dynamics. Fluorescent proteins (FPs) as intrinsic intracellular reporters have many advantages over conventional fluorescent dyes in live cell imaging. They are genetically encoded, therefore, no loading of dye is necessary, thereby enabling noninvasive imaging. The FPs can be precisely targeted to almost any organelle, compartment, or tissue. Moreover, FPs have been designed to respond to a greater variety of biological events and signals than conventional dyes. The sensors based on FPs have been used in living cells to report cellular concentrations of ions, second messengers cAMP and inositol phosphates, ATP, redox potential, reactive oxygen species, enzyme activities, and pH (review [5]).

**Figure 1.**
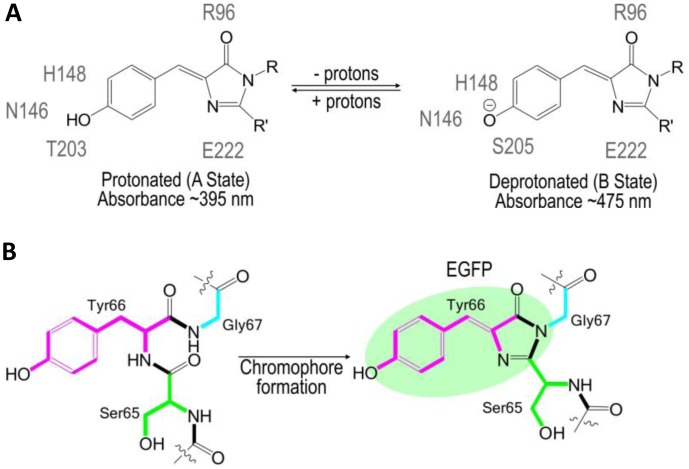
(**A**) A two state model of pH dependent ground states of wtGFP. Four molecules of water and side-chains of amino acids depicted in grey are involved in hydrogen-bonding with the chromophore. (**B**) Chromophore formation involves cyclization, imidazolinone ring system formation, dehydration and oxidation.

The use of FPs began with the identification and subsequent characterization of wild-type green fluorescent protein (wtGFP) from the jellyfish *Aequorea victoria* [5,6]. Within the β-barrel structure of GFP, three consecutive amino acids S65, Y66, G67 form by post-translational cyclization a chromophore that, by itself, is not fluorescent. Amino acids Q69, Q94, R96, H148, T203, S205, and E222 that are placed around the chromophore are necessary for GFP fluorescence [7]. These residues function as proton donors and acceptors and define spectral and photophysical properties of GFPs [8]. Bizzarri *et al.* [9] provide a detailed review of the physical and chemical characteristics of the chromophores of different GFP mutants. Briefly, the optical properties of protonated-neutral and deprotonated-anionic states differ among FPs. Among other parameters, pH also affects the equilibrium between protonated and deprotonated forms. Some FPs show pH-dependent absorption/ fluorescence spectra in the physiological pH range and are, therefore, used as pH sensors for live cell imaging (Figure 1).

Further, pH-sensitive FPs are divided in two groups: (i) intensity-based sensors and (ii) ratiometric pH-sensitive proteins with dual excitation and/or emission spectra. There is also a third group of pH-sensitive proteins—chimeric proteins, which are concatenated pH-sensitive FPs with another fluorescent or luminescent protein that gain novel pH-dependent spectral properties through fusion. Some of the most frequently used pH sensors are described below.

## 2. Intensity-Based pH Indicators

The protonated state of the wtGFP absorbs at 395 nm and emits at 508 nm, while the deprotonated state absorbs at 475 nm and emits at 503 nm [9]. The neutral, protonated state is progressively converted to the anionic, deprotonated state as the pH increases. The so-called intensity-based, nonratiometric pH sensors possess good pH responsiveness, but are characterized by very poor emission from the neutral chromophore (395-nm excitation). Due to the rather complex calibration of the intensity-based pH sensors, they are usually used to report changes in pH_i_ rather than pH_i_ itself. Many variations of pH-sensitive FPs have been created with random and targeted mutagenesis of the *Aequorea* wtGFP [5] or the *Discosoma* mRFP [10]. These FPs differ in terms of apparent pK_a_ that defines an operational pH range of the sensor and by excitation/emission spectra that delineate the equipment used for analysis (Table 1).

Kneen *et al.* [11] were among the first to explore the possibility that the GFP could be used as a pH sensor in living cells. They synthesized four green GFP mutants with pK_a_ values 6.0 (EGFP, GFP-F64L/ S65T), 5.9 (S65T), 6.1 (Y66H), and 4.8 (T203I). The absorbance and emission spectra of two S65T mutants are similar to that of the wtGFP with two absorption maxima at 390 and 490 nm, with the only excitation wavelength at 490 nm and emission at >510 nm. To evaluate the suitability of enhanced GFP (EGFP) as a pH sensor in living cells, the EGFP was expressed in cytosol, mitochondria, Golgi, and the endoplasmic reticulum of CHO and LLC-PK1 cells. Two other mutants (Y66H and T203I) have blue-shifted spectra with single maxima and parallel pH-dependent changes in absorbance and fluorescence [11]. In the same year, another blue-shifted pH sensitive protein was synthesized. Extensive mutagenesis of wtGFP generated an enhanced cyan GFP (ECFP); its absorption and emission spectra peak at 440 nm and 480 nm, respectively, with pK_a_ 6.4 [12].

Independent of Kneen *et al.*, Miesenbock *et al.* [13] generated a GFP mutant—termed ecliptic pHluorin—with pK_a_ 7.1. Substitutions S147D, N149Q, T161I, S202F, Q204T, and A206T on wtGFP provoked a reduction in 395 nm excitation and an increase in 475 nm excitation, which is induced by a pH shift. During acidification, the e-pHluorin gradually loses fluorescence. The e-pHluorin is widely used for imaging vesicle fusion events because it is nonfluorescent at a pH lower than 6 at 475 nm excitation, but still weakly seen at 395 nm. A brighter version of the e-pHluorin, a superecliptic pHluorin (e_s_-pHluorin) [14] contains two additional mutations, F64L and S65T, in the original ecliptic pHluorin, thereby leading to enhanced fluorescence with similar absorption/emission spectra. These two mutations are also characteristic of EGFP [11]. A plant ecliptic pHluorin—PEpHluorin [4]—is a fusion between a soluble-modified GFP (smGFP) [15] with F99S mutation and e-pHluorin with additional mutations M153T and V163A [4]. The presence of smGFP decreases pK_a_ to 6.0 with an acidification-dependent decrease at excitation peaks 395 nm and 475 nm, recorded at 512 nm.

An enhanced yellow fluorescent protein (EYFP) has a yellow-shifted absorption and fluorophore emission with respect to conventional green FPs [12,17]. This shift in the spectra is the result of a T203Y substitution. Further, EYFP shows an acidification-dependent decrease in the absorbance peak at 514 nm, an emission peak at 527 nm, and a concomitant increase in the absorbance at 390 nm, which is nonfluorescent. An improved yellow FP mCitrine was generated by Q69M and V68L mutations to EYFP. These substitutions reduce sensitivity to chloride ions, improve maturation at 37 °C, and lower pK_a_ to 5.7 [18,19]. The more acidic pK_a_ classify mCitrine as a pH sensor suited for pH analysis of *medial/trans*-Golgi, as well as secretory and endocytic organelles [12].

Abad *et al.* [20] developed a sensor named mtAlpHi for analysis of mitochondrial alkaline pH with an apparent pK_a_ of approximately 8.5. The sensor is composed of EYFP taken from camgaroo-2 [18]. A calmodulin from camgaroo-2 is replaced with aequorin, comprising 73 amino acids that contain second and third EF-hand domains. mtAlpHi undergoes changes only in fluorescent intensity upon pH changes; it is prone to potential artifacts of all nonratiometric indicators. However, the co-transfection with mtECFP largely circumvented this drawback.

Some pH-sensitive indicators have an excitation and emission shifted toward red spectra. These red-shifted FPs are particularly practical for multicolor labeling in combination with green or yellow FPs. Thus far, only pHTomato [24] and mNectarine [25] have been used for pH analysis in living cell imaging. The emission intensities of pHTomato are highly pH-dependent with a pK_a_ of 7.8. Along the mtAlpHi [20], this is the highest determined pK_a_ among intensity-based pH sensors, thereby making the sensor suitable for following mitochondrial pH [24]. The pH influences the fluorescence intensity of pHTomato, but not the excitation and emission waveforms. Conversely, the absorbance spectra of mNectarine [25] recorded at various pHs reveal complex changes in profile that appear to include a mixture of anionic cyan-absorbing (489 nm) and orange-absorbing (558 nm) forms of the protein at high pH, and a mixture of protonated violet-absorbing (387 nm) and blue-absorbing (453 nm) forms of the protein at low pH [25]. The apparent pK_a_ of mNectarine is 6.9, with excitation at 558 nm and emission measured at 578 nm.

Recently, a new generation of pH-sensitive FPs has been developed with pK_a_ values ranging from 5.3 to 6.5; however, they have not been tested as pH sensors in live cell experiments. The absorption/excitation spectrum for green FP mWasabi is narrower than that of EGFP with the same emission spectra; therefore, less cross-talk is expected when used in combination with blue- and cyan-shifted FPs [16]. The Clover is very bright yellow FP, with an acidic pK_a_ of 6.2, thereby making it suitable for imaging pH changes of Golgi-network and secretory vesicles [21]. The FPs derived from the *Discosoma* mRFP are mainly red-shifted [23–26]. Further, Shaner *et al*. [22] developed two highly photostable mOrange variants by random and iterative mutagenesis. mOrange M163K and mOrange2— with pK_a_ 7.5 and 6.5, respectively—exhibit enhanced photostability, efficient maturation, and increased pH sensitivity [22,23]. The other pH-sensitive red-shifted FPs are mRuby2, mKate2, mTangerine, and mKate. Their pK_a_ varies from 5.3 to 6.2, which makes them suitable for imaging pH changes from <5.0 to 6.5 [21,26,27]. For example, the maturation of endosomes coincides with a pH reduction from 6.8 to <5.5 [1,28,29]. A drawback for mKate, mRuby2, and mTangerine pH sensors is that they are not very bright fluorophores with low quantum yield compared to other FPs. The problem can be elegantly solved by tethering two FPs, as done for mOrange [23].

## 3. Ratiometric Sensors Constituted by a Single Fluorescent Protein

The emission intensity of intensity-based pH indicators depends on the total concentration of fluorophore; therefore, it is difficult to determine whether observed changes in fluorescence are due to pH change or indicator concentration. An elegant way to circumvent the problem of the intensity-based pH indicators are ratiometric pH indicators that do not require an independent means of measuring the concentration of protein. Dual excitation ratiometric pH sensors have bimodal excitation spectra, and some also have dual-emission spectra with dose-dependent changes in excitation/emission at increasing acidity (Table 2). These features are an important advantage over the attributes of a single-wavelength FPs, such as e-pHluorin, because they make ratiometric sensors resistant to photo-bleaching and provide variability of indicator loading.

The first study on ratiometric GFP by excitation was that of Miesenbock *et al.* [13]. Ratiometric GFP also called ratio-pHluorin (r-pHluorin), and displays an increase in fluorescence at 508 nm after a 475-nm excitation concomitantly with a decrease after a 395-nm excitation upon a pH shift from 7.5 to 5.5, with apparent pK_a_ of 6.9. The functionality of r-pHluorin has been initially tested expressed in *trans*-Golgi network, endosomes, and synapses. Subsequently, a modification to r-pHluorin was introduced to enhance the brightness of pHluorin, which was achieved with additional mutations F64L and S65T [30], which are characteristic for EGFP [11]. pHluorin2 is used for pH analysis in human cell lines and yeast *Saccharomyces cerevisiae* [30,31]. Further, a M153R mutation in the r-pHluorin increases the brightness of e-pHluorinM153R and its *in vivo* stability, while it does not affect the 410/470 nm excitation ratios at various pH values [32]. The r-pHluorin with mutation A206K named RaVC has been used to study the pH_i_ of the filamentous fungus *Aspergillus niger* [33,34]. A plant dual-excitation ratiometric pHluorin, PRpHluorin—which is similar to PEpHluorin [4]—is a fusion between a soluble modified GFP (smGFP) [15] with F99S mutation and pHluorin with additional mutations M153T and V163A [4]. The presence of smGFP decreases pK_a_ to 6.6 with no effect on the spectral waveforms of pHluorin.

Hanson *et al*. [35] introduced four ratiometric sensors named deGFP1 (S65T, H148G, and T203C), deGFP2 (S65T, C48S, and H148C), deGFP3 (S65T and T203C), and deGFP4 (S65T, C48S, H148C, and T203C) that are ratiometric by emission. The deGFPs have a pK_a_ ranging from 6.8 to 8.0. With acidification, with the excitation of deGFPs at 400 nm, a green emission (peak at 515 nm) converts to a blue one with a peak at 460 nm. In addition, the anionic chromophore is directly excited at 480–500 nm, and only a green emission is detected. Therefore, the pH-dependent dual emission is defined at 460 and 515 nm for excitation of a neutral chromophore at 400 nm. The deGFPs are very promising for bioimaging applications, particularly for two-photon excitation.

E^2^GFP, a GFP (F64L, S65T, T203Y) variant [36], belongs to the yellow FP class and displays ratiometric characteristics similar to deGFPs [35]. The E^2^GFP is an excellent ratiometric sensor by excitation (e.g., λ_x_ 458 and 488 nm; λ_e_ 500–600; pK_a_ 6.9) and by emission (λ_x_ 458 nm; λ_e_ 475–525 and 515–600 nm; pK_a_ 7.5). The sensor is quenched by halide ions, but the ratiometric measurements are not affected due to its ratiometric characteristics. Subsequently, E^1^GFP [37] was developed; it is spectroscopically similar to E^2^GFP, but less sensitive to halide ions and with a pK_a_ ranging from 6.4–6.6; therefore, it is more tailored for mildly acidic intracellular compartments. The fluorescence spectra are consistent with the presence of two pH-interconverting forms. Excitation at 408 and 473 nm yields a pH-dependent emission spectra with a peak at 508–510 nm, which is red-shifted (peak at 523 nm) as the pH increases. Further, for live cell imaging, two excitation-wavelength ratiometric setups were used: excitations 488 nm and 458 nm or 488 nm and 406 nm with an emission interval of 500 to 600 nm.

The sea cactus *Cavernularia obesa* GFP (CoGFP)—like deGFP and E^n^GFP—has pH-sensitive, ratiometric dual-excitation/emission properties, and dual-color emission maxima upon single-wavelength excitation [38]. The excitation at 388 nm leads to blue fluorescence with a peak at 456 nm at pH 5 and below, and green fluorescence with a peak at 507 nm at pH 7 and above. Excitation at 498 nm triggers green fluorescence with a peak at 507 nm from pH 5–9. Further, fluorescence at 507 nm is ratiometric to that at 456 and 388 nm excitation, with pK_a_ 6.5. Additional mutations to wtCoGFP generated either brighter fluorescence or different spectral characteristics. It must be noted that only variant-0 has been tested further in live cell imaging.

Shulte *et al.* [39] developed a novel GFP variant isolated from the orange seapen *Ptilosarcus gurneyi* with good pH responsiveness and excellent dynamic ratio range. The *Pt*-GFP is ratiometric by excitation; however, the pH dependency of the fluorescence spectra is just opposite to that of pHluorin. The *Pt*-GFP with pK_a_ 7.3 displays an increase in 540-nm fluorescence after a 390-nm excitation concomitantly with a decrease after a 510-nm excitation upon a pH shift from 7.5 to 5.5. The *Pt*-GFP sensor is also stable at a pH below 5, which is an advantageous feature when organelles with low pH are labeled.

Thus far, only one red-shifted ratiometric pH sensor has been identified. Tantama *et al.* [40] engineered a ratiometric pH sensor, pHRed by mutagenesis of the long Stokes-shifted fluorescent protein mKeima. The peak fluorescence emission of pHRed occurs at 610 nm. The acidification from pH 9 to 6 causes a 7-fold increase in the 585 nm peak intensity and a 4-fold decrease in the 440 nm peak intensity; both peaks respond with a pK_a_ of 7.8. Moreover, pHRed exhibits a pH-dependent fluorescence lifetime that makes the pHRed suitable for imaging intracellular pH by fluorescence lifetime imaging microscopy (FLIM).

SypHer [41], a ratiometric circularly permutated YFP HyPer [42] with C199S mutation, is the only ratiometric pH sensor with a pK_a_ of above 8. The pK_a_ of 8.7 permits accurate measurements of mitochondrial pH. The fluorescence intensity at 535 nm increases markedly with pH at an excitation wavelength of around 490 nm and mildly decreases at an excitation wavelength of 405 nm.

## 4. Other genetically Encoded pH Sensitive Sensors

By joining two or more FPs with different pH-dependency of fluorescence spectra, a new chimeric FP with novel excitation/emission spectra and altered pH-dependency is synthesized (Table 3). This approach is mainly used to convert intensity-based FPs to ratiometric pH sensors. Using a synthetic biology approach, we can generate numerous combinations of FPs and tailor the FPs to our requirements.

Three novel ratiometric pH sensitive sensors GFpH, YFpH [43], and pHERP [44] were obtained by fusing GFPuv, a low-pH sensitivity mutant excitable at 380 nm, with an intensity-based pH sensitive GFP, EGFP, or EYFP. The YFpH and pHERP sensor are both fusions of GFPuv and EYFP, with changed order of named FPs. When excited at 380 nm and 480 nm, the GFpH emits at 510 nm. The pK_a_ of GFpH is 5.6 (380 nm excitation) and 6.2 (480 nm excitation) and the apparent pK_a_ is 6.2. The fluorescence maximum of YFpH and pHERP is 527 nm when excited at 480 nm and the apparent pK_a_ is 6.5. Upon excitation at 380 nm, the emission of YFpH and pHERP shifts from 527 nm to 506 nm with the acidification.

Arosio *et al.* [45] developed the ratiometric sensor ClopHensor that is suited for real-time optical detection of chloride ions and pH in live cells. The ClopHensor exploits the spectral and chemical characteristics of the E^2^GFP anion-binding site [36] that is linked to the DsRed, a fluorescent protein, which is insensitive for protons and chloride ions. With tethering E^2^GFP to DsRed, the pH-sensitive spectral characteristics of E^2^GFP remain unchanged. Mutations H148 and V224L of ClopHensor shift pK_a_ to more alkaline values and improve affinity for chloride ions [46].

pHusion [47] is composed of a tandem concatenation of EGFP and mRFP1, and also function as a ratiometric pH sensor. To obtain a 1:1 stoichiometry, the two fluorescent proteins are fused by a short peptide linker, permitting for ratiometric measurements of pH changes, where mRFP1 functions as an intramolecular reference.

The concatenation of pH-sensitive with pH-insensitive FPs can be used for the combination of virtually any FPs, assuming that the excitation and emission spectra of concatenated FPs do not overlap. Such fusions are sensitive for photo-bleaching; moreover, more sophisticated equipment is required due to multi-excitation/emission spectral properties.

## 5. pH in Cellular Organelles

**Figure 2.**
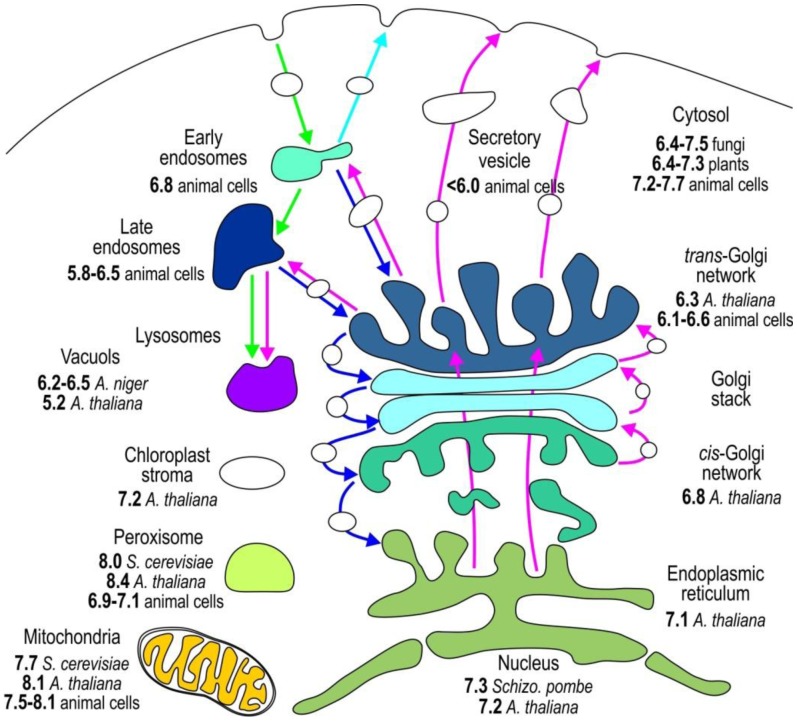
The pH of individual subcellular compartments in a prototypic eukaryotic cell. We show only pH values obtained using the genetically encoded pH-sensitive FPs as indicators. The pH values are collected from different sources and are referenced in Table 4.

The compartmentalization of the eukaryotic cell provides distinct environmental conditions for the optimal operation of individual metabolic pathways. The function of individual organelles depends on the stringently regulated pH (Figure 2) because protons dictate the charge of macromolecules and generate transmembrane electric potential [1,3]. To study pH_i_ *in vivo*, accurate pH determination is essential. In this section, we outline the genetically encoded sensors applied to monitor the pH of individual subcellular compartments (Table 4).

Most organelles, except nucleus, have their own specific pH value associated with the processes that occur in these compartments. The nuclear membrane has an abundance of pores that are a weak barrier for protons; therefore, nuclear pH (pH_N_) is similar to that of the surrounding cytosol. Both cytosolic pH (pH_C_) and pH_N_ of various resting cells are slightly above neutral, between 7.2 and 7.7 (Figure 1, and see Tables 4, 5, 6 and 7 for references) [11,12,33,34,36,39,40,44,46,48]. To specifically relocate the pH sensor to the nucleus, a nuclear localization signal NLS is fused to pHluorin [4,49]. A cytosolic sublocation of pHluorin tethered with palmitoylation site, caveolin-1, α1B-adrenoreceptor, or hCTN3 revealed that the pH of these cytosolic microdomains is not uniform and is 7.3, 7.3, 6.8, or 6.5–7.5, respectively [25,43,46,50,51]. The pH_C_ of yeasts *S. cerevisiae*, *Candida glabrata*, and *Schizosaccharomyces pombe* is highly influenced by environmental changes, such as nutrients and external pH. Their pH_C_ differs from 6.4 when cells are starved to 7.5 when glucose is present in the media [31,49,52–55]. The pH_C_ is also not uniform throughout plant tissues. The pH_C_ of *A. thaliana* varies from 6.4 at the root cap to 7.3 in meristem cells, thereby proving the presence of significant pH gradients among different developmental regions in plant roots [4,15,47].

The pH sensors targeted to the lumen of endoplasmic reticulum via retrograde transport based on KDEL receptor show that the pH of endoplasmic reticulum (pH_ER_) is very close to its pH_C_ [4,11,56]. The luminal pH throughout the secretory pathway becomes progressively more acidic (Figure 2, Table 5). The *cis*-Golgi with pH 6.8 is more acidic than the endoplasmic reticulum [4], and the acidification becomes more apparent when the pH changes from 6.1 to 6.6 in the *trans*-Golgi network. Rivinoja *et al*. [57] used GT-EGFP to show that the *medial/trans*-Golgi pH in cancer cells MCF-7 and HT-29, SW-48, is significantly more alkaline (pH 6.75) than in control cells (pH_G_ 5.9–6.5), thereby clearly emphasizing the importance of stringent pH homeostasis. Tagging subcellular domains of Golgi is rather difficult. Many Golgi proteins reside in endoplasmic reticulum for a short period of time, thereby indicating that proteins normally cycle between the Golgi and endoplasmic reticulum [58]. Furthermore, some Golgi proteins such as TGN38/46, a *trans*-Golgi network (TGN) protein [59], and GPP130, a *cis*-Golgi protein [60], cycle between the Golgi and endosomes. Directing the pH sensor to *medial/trans*-Golgi is achieved with N-terminal linking pHluorin to β-1, 4-galactosyltransferase [11,12,57,61], 2,6-sialyltransferase [44], and TGN38 in a *trans*-Golgi network [59]. ManI-PpHluorin—pHluorin fused with glycine max mannosyl-oligosaccharide 1,2-alpha-mannosidase—is used to specify the *cis*-Golgi luminal pH [4].

Due to the heterogeneity of secretory vesicles and their acidification during secretion, the analysis of vesicular pH presents a serious challenge (Table 6). Primarily, e-pHluorin is used for the analysis of vesicular dynamics; therefore, spatial and temporal pH changes rather than the pH of recycling vesicles are known. Synaptic vesicles maintain an acidic lumen with a resting pH value of 5.6 [13]. Therefore, instead of pHluorins, FPs with a pK_a_ of almost 6 might be better suited for pH measurements of acidic subcellular compartments, including maturing endosomes. During the so-called process of de-granulation, lytic granules and synaptic vesicles release their content when fusing to the plasma membrane [62]. The dynamics of secretory vesicles have been studied using DsRed-FasL-e_s_-pHluorin for visualizing fusion pore openings [62], VGLUT1-2xmOrange2, VGLUT1-pH, and synapto-pHluorin and SytIV-pHluorin for monitoring re-acidification of synaptic vesicles after endocytosis [11,13,14,23,24,63–66]. Since synaptophysin displays less background expression on the plasma membrane than synaptobrevin/VAMP2, synaptophysin is used for an improved sypHTomato reporter of vesicular turnover [24]. Dean *et al*. [67] used e-pHluorin to report the location and pH of organelles to which the synaptotagmin isoforms are targeted. Among 15 isoforms, only syt-1 and 2 are targeted to synaptic vesicles; the other 13 are located in dendrites and axons.

Spatial and temporal pH changes have been analyzed for many specialized vesicles, such as insulin secretory granules [68], large dense-core vesicles [45] and maturing endosomes [4,50,59]. pHluorin, ECFP, and E^1^GFP are targeted to vesicles through fusing the pH sensor to luminal site of proteins that are characteristic of those vesicles [4,30,50,56,59,68,69].

Peroxisomes [73], organelles that derived from endoplasmic reticulum, are involved in many different cellular functions, such as breakdown of fatty acids, polyamines and D-amino acids. r-pHluorin is directed to peroxisomes with peroxisomal targeting SKL/SRL peptide, and is used to measure peroxisomal pH, which range from 6.9 to 8.4 [4,55,70] (Table 7). A chitinase and an N-terminus of Rubisco activase (recA) protein fused to pHusion and PRpHluorin directed chimeric proteins to apoplasts and plastid stroma of *Arabidopsis thaliana*, respectively [4,47]. The pH of the cell cortex of *Drosophila* S2 cells was analyzed using pHMA sensor—pHluorin tagged to moesin [71].

Recent studies on mitochondrial pH present a good demonstration of the importance of the selection of a pH sensor that matches the pH of the subcellular compartment [74] (Table 7). The resting mitochondrial matrix pH (pH_M_) is markedly alkaline from 7.6 to 8.1 [4,11,12,20,40,41,55,72]. Previously, the pH sensors pHluorin, ECFP, EYFP, and ECFP with a pK_a_ of approximately 7 have been used to measure pH_M_ [11—13,74]. These sensors are insensitive to small pH changes at pH_M_; therefore, pH_M_ dynamics are easily missed. Recent improvements in live cell fluorescence imaging have revealed that proton concentration rapidly fluctuates within individual mitochondria, as determined using mitoSypHer [39,75]. The pK_a_ of ratiometric mitoSypHer is approximately 8, which is similar to the pK_a_ of ratiometric pHRed [40] and intensity-based mtAlpHi [20] and pHTomato [24]—all of which are well-suited for pH_M_ measurements.

## 6. Instrumentation

The intrinsic pH of studied subcellular compartments and the type of available equipment determine the selection of FPs for live cell imaging. Intensity-based pH sensors require a collection of data at one wavelength; therefore, rather basic equipment is necessary for one-wavelength intensity measurements. Conversely, a collection of fluorescence at two wavelengths is necessary for dual emission pH sensors; thus, instruments with multiple detectors and filters are needed. Dual excitation sensors, like ratiometric pHluorins, are analyzed with advanced equipment with two excitation sources. Moreover, the spatial analysis of pH within organelles is greatly benefitted with equipment that has a good resolution in Z-direction. Therefore, confocal microscopy is favored over wide-field fluorescent microscopy. The recent developments of highly effective imaging setups with improved resolution and speed, as well as supercontinuum laser have greatly improved the detection of pH in cultured cells and living organisms.

A traditional fluorescence spectroscopy is the most extensively applied technique for analyzing the pH_i_ of yeast when pHluorin is used as a pH sensor [51–54]. Although spectroscopy rapidly records the average pH_i_ of a population, it provides insufficient single-cell information.

**Figure 3.**
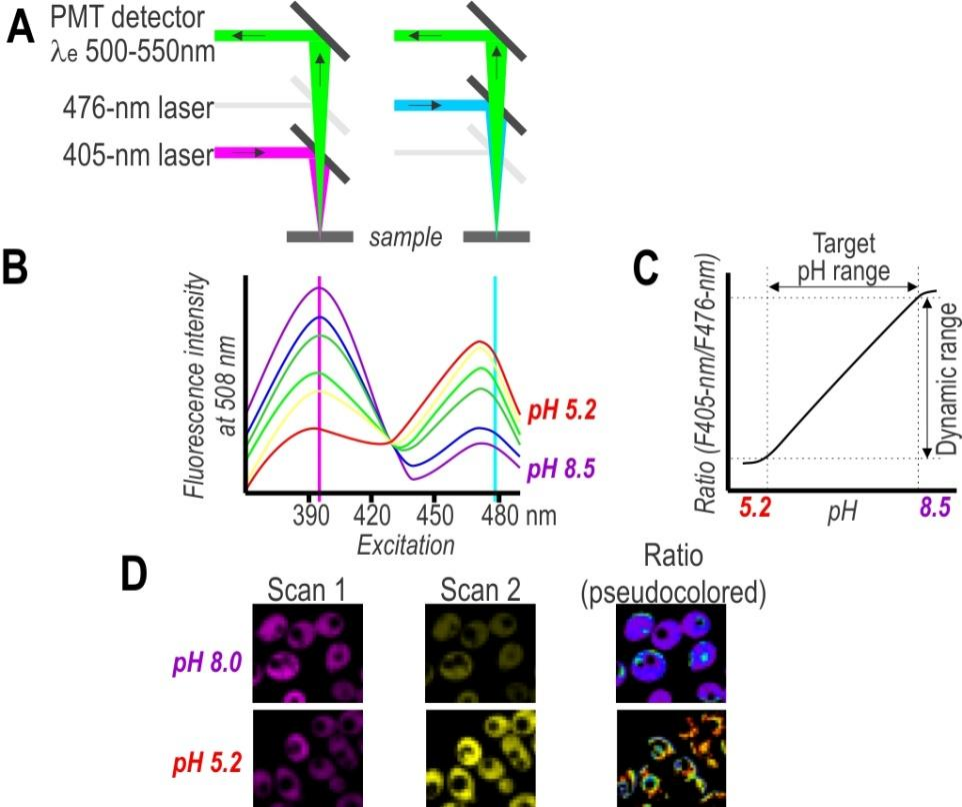
(**A**) A set up of dual excitation sequential scanning confocal microscope for imaging ratiometric pHluorin probes. (**B**) Fluorescence excitation spectra of pHluorin in buffers with pH ranging from 5.2 to 8.5. (**C**) A hypothetical pHluorin dose reponse curve; a plot of fluorescence ratio *versus* pH. The ratios between the emission intensities at 405- and 476-nm are calculated. The physiological target pH range and physiologically relevant dynamic range are indicated. (**D**) Pseudocolored image (right) of permeabilized yeast cells in buffered solution with nigericin is calculated from images taken at 405- and 476-nm excitation (Scans 1 and 2). Different colors are assigned to defined pH values in accordance with the *in situ* calibration curve (blue, alkalinic; red, acidic). The *in situ* calibration curve is shown in panel D.

On the other hand, single-cell analysis offers more detailed insight into population variability, thereby facilitating a considerably deeper understanding of cell physiology. To obtain spatial distribution and a detailed mapping of the pH in single cells, researchers have used pH-sensitive FPs in combination with fluorescence microscopy (Figure 3). Despite the advantages of this approach, the primary drawback of this technique is the need to post-process images for evaluating subpopulation content. Nonetheless, the development of confocal microscopy revolutionized biological sciences and provided great tools for cellular imaging on a subcellular level. Thus, confocal microscopy with excellent resolution (xy 0.2–0.5 µm and z 0.6–1.5 µm) provides a very effective way to obtain high-resolution pH maps.

Historically, equipment dictated the evolution of fluorescent dyes and FPs. Therefore, cyan, green, and yellow FPs whose excitations correspond to the emission lines of the most widespread Argon laser are best studied (Table 1). Subsequently, microscopes equipped with violet and red laser along with Argon laser promoted the evolution of red- and far red-shifted FPs, as well as ratiometric pH sensors by excitation (Table 2). Advanced confocal microscopes equipped with multiple independent detectors permit simultaneous collection of several fluorescence signals, which agrees with ratiometric sensors by emission (deGFP [35], E^n^GFP [36,37], Wt-CoGFP [38], and pHusion [47]) and multicolor labeling. Multiphoton microscopy in cases of low photo-toxicity and photo-bleaching is an alternative to single-photon excitation confocal microscopy. The deGFPs and E^n^GFPs have the potential of two-photon ratiometric indicators [35–37,48]. Further, fluorescence life time microscopy (FLIM) offers an alternative solution for life cell imaging. pHRed [40] and ECPF [69] exhibit a pH-dependent fluorescence lifetime that can be used to image intracellular pH.

**Figure 4.**
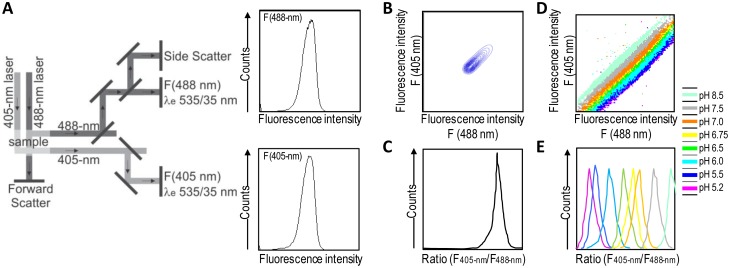
Flow cytometry for high-throughput analysis of cytosolic pH in cells expressing ratiometric pH sensors by excitation [31]. (**A**) The light paths of excitation at 405 and 488 nm and the emission optics are illustrated. Fluorescence intensity data are depicted for both detection channels—F(488 nm) and F(405 nm). (**B**) A 2D dot plot of F405-nm *versus* F488-nm shows grouped fluorescence signals. (**C**) The ratio of the fluorescence intensities of F(405 nm)/F(488 nm) plotted as the ratio height signal generates a narrow peak. (**D**) An overlay of 2D dot plots of F(405 nm) *versus* F(488 nm) for pH sensor at indicated pH values. (**E**) An overlay of ratios of the fluorescence intensities of F(405 nm)/F(488 nm) depicted from (**F**). Note: In less than 2 min, the fluorescence signals of 50,000 cells can be analyzed and post-processed.

A good alternative to spectroscopy and microscopy is flow cytometry, which combines rapid high-throughput, analysis, and acquisition of multiparameter results at the single-cell level for each cell in a population. For the dual excitation of all pHluorin-based ratiometric pH probes, a flow cytometer equipped with split optics for 405- and 488-nm light paths is necessary [31,55] (Figure 4). After excitation with 488-nm and 405-nm laser light, green fluorescence is detected in separate channels, and the ratios of two fluorescence parameters (*i.e.*, F405-nm and F488-nm) are calculated for every cell by dividing the emission signals. Finally, to correlate ratios with pH, a calibration curve that links fluorescence intensity ratios to pH is generated.

Ratiometric flow cytometry goes beyond microscopy for high-throughput analysis and screening with automatic post-processing of obtained data. Conversely, it does not permit the monitoring of particular individual cells over time, thereby making it a complementary solution rather than a replacement for microscopy techniques.

## 7. Conclusions

Undoubtedly, the maintenance of an appropriate pH within individual membrane-enclosed compartments is important for normal physiology of the cell and organelles. Over the previous decade, considerable progress has been made in understanding the pH regulatory mechanisms of the cell that progress in tandem with recent developments of highly effective imaging setups and genetically encoded pH-sensitive fluorescent sensors. In merely fifteen years, we have witnessed an expansion in genetically encoded pH-sensitive fluorescent proteins. pH sensors span almost the entire visible spectrum from green to red and with pK_a_ that extends over the entire physiological pH range. Most modern FPs have been modified by random and/or iterative mutagenesis to optimize, improve, or enhance fluorescence characteristics and their stability as proteins. The sensors for virtually any subcellular location have been engineered from only a few building blocks. The interplay of sophisticated imaging techniques and transgene pH sensors has provided a very effective method for obtaining spatial and temporal high-resolution pH maps and the ability to monitor signaling dynamics on single-cell and subcellular levels.

In this review, we have described pH-sensitive fluorescent proteins that we consider to be useful or as having potential for generating novel sensors for live cell applications. The trend in the evolution of pH sensors is oriented toward generating brighter, photostable yellow and red ratiometric FPs to limit the cell damage that might be caused by the illumination of the fluorophore. Advances have been made for pH-sensitive FPs with narrow excitation and emission peaks to suit multicolor labeling. The narrow spectral peaks minimize the crosstalk between fluorophores, thereby providing more accurate results in multicolor analysis. With regard to developments in equipment, pH sensors that are suited for fluorescence lifetime microscopy and two-photon laser scanning fluorescence microscopy have been engineered. Transgene pH sensors are already changing the boundaries of our understanding of *in vivo* cellular pH dynamics at scales from the subcellular to the entire organism. Further, pH-sensitive FPs expressed in the cell and subcellular compartments provide the most physiologically relevant information on the spatial and temporal behavior of pH homeostasis. Overall, we anticipate that this overview will help in deciding which pH-sensitive fluorescent protein should be selected for forthcoming cellular imaging experiments, and how the selected pH sensor could be applied to obtain accurate answers to various questions in this regard.

## Figures and Tables

**Table 1.** Intensity-based pH-sensitive fluorescent proteins.

	**pK_a_**	**λ_x1_(nm)**	**λ_x2_(nm)**	**λ_e_(nm)**	**Quantum Yield**	**Reference**

		**ε (mM^−1^·cm^−1^)**				
Green FP						

ECFP (GFP-K26R, F64L, S65T, Y66W, N146I, M153T, V163A, N164H, H231L)	6.4	b	440	480	0.40	[12]
			33			
EGFP (GFP-F64L, S65T, H231L)	5.8	396^a^ 29	489	509	0.60	[11,12]
			60			
Ecliptic pHluorin (GenBank: AF058695) (GFP-S147D, N149Q, T161I, V163A, S175G, S202F, Q204T, A206T)	7.1	396^a^	476	508	b	[13]
Superecliptic pHluorin (GenBank: AY533296) (e-pHluorin-F64L, S65T)	7.2	396^a^	476	508	b	[14]
PEpHluorin	b	395^a^	475	512	b	[4]
mWasabi (GenBank: EU024648)	6.5	b	493	509	0.80	[16]
			90			

Yellow FPs						

EYFP (GFP-F64L, S65G, S72A, T203Y, H231L)	7.1	390^a^	514	527	0.61	[12,17]
			84			
mCitrine(GFP-F64L, S65G, V68L, Q69M, S72A, T203Y, H231L)	5.7	b	516	529	0.76	[18,19]
			77			
mtAlpHi (S65G,V68L, Q69K, S72A, T203Y=Camgaroo2 calmodulin replaced with short aequorin)	8.5	b	498	522	b	[20]
Clover	6.2	b	505	515	0.76	[21]
			111			

Red FPs						

mOrange2 (GenBank: DQ336159) (mOrange-Q64H, F99Y, E160K, G196D)	6.5	b	549	565	0.60	[22,23]
			58			
pHTomato (GenBank: JQ966306) (mStrawberry-F41T, Q66T, F83L, S182K, I194K, V195T, G196D)	7.8	b	550	580	b	[24]
mNectarine (GenBank: FJ439505)	6.9	b	558	578	b	[25]
			58			
mKate (GenBank: EU383029)	6.2	b	588	635	0.28	[26]
			32			
mKate2 (GenBank: JB331973)	5.4	b	588	633	0.40	[26]
			63			
mTangerine (GeneBank: AY678270)	5.7	b	568	585	0.30	[27]
			568			
mRuby2	5.3	b	559	600	0.38	[21]
			43			

a Poor or no emission at this wavelength excitation;

b not determined/not applicable.

**Table 2.** Ratiometric pH-sensitive fluorescent proteins.

	**pK**	**λ_x1_(nm)**	**λ_x2_(nm)**	**λ_e1_(nm)**	**λ_e2_(nm)**	**Reference**

		**ε (mM^−1^·cm^−1^)**				
pHluorins						

R-pHluorin (GenBank: AF058694) (GFP-E132D, S147E, N149L, N164I, K166Q, I167V, R168H, L220F)	6.9	395	475	508^a^	b	[13]
R-pHluorin2 (R-pHluorin-F64L, Q80R, E132D, S175G)	7.1	395	475	509^a^	b	[30,31]
R-pHluorin(M153R) (R-pHluorin-M153R)	7.1	395	475	509^a^	b	[32]
pHGFP	b	410	470	535^a^	b	[15]
PrpHluorin (R-pHluorin for plants)	6.6	395	475	515^a^	b	[4]
RaVC (R-pHluorin-XX)	7.1	395	476	508^a^	b	[33,34]

deGFP						

deGFP1 (S65T,Q80R, H148G, T203C)	8.0	400	504	466	516	[35]
deGFP2 (S65T, C48S, Q80R, H148C)	7.3	398	496	462	517	[35]
deGFP3 (S65T, Q80R, T203C)	6.9	396	508	461	518	[35]
deGFP4 (S65T, C48S, Q80R, H148C, T203C)	7.4	400	509	462	518	[35]

E^n^GFP						

E^2^GFP (GFP-F64L, S65T, T203Y, L231H)	6.9–7.5^e^	423/401^d^	515	510/523^d^	b	[36]
		*32/23*	*22*		
E^1^GFP (GFP-F64L, T203Y)	6.4–6.6^e^	410/400^d^	509	510/523^d^	b	[37]
		*31/29*	*5*			

Others						

Wt-CoGFP	6.5^e^	388	498	456/507^d^	507	[38]
*Pt*-GFP	7.3	390	502	508^a^	b	[39]
pHRed (mKeima-A213S)	7.8; 6.9^c^	440	585	610^a^	b	[40]
SypHer (HyPer-C199S)	8.7	420	490	535^a^	b	[41]

a Valid for both excitations (λx1 and λx2);

b not determined/not applicable;

c pK determined with FLIM;

d at pH<5/at pH>8,

e pK depends on combination of excitation/emission set up.

**Table 3.** pH-sensitive fluorescent proteins.

	**pK**	**λ_x1_(nm)**	**λ_x2_(nm)**	**λ_e1_(nm)**	**λ_e2_(nm)**	**Reference**
GFpH GFPuv-EGFP	6.1	380	480	510	a	[43]
YfpH GFPuv-EYFP	6.5	380	480	509/527	a	[43]
pHERP EYFP-GGGLEDPRVPVEK-GFPuv	6.5	397	515	520	a	[44]
ClopHensor E^2^GFP-linker 20 aa-dsRedm	6.8	458 and 488	543 for DsRed	590	630 for DsRed	[45]
ClopHensor (H148G,V224L)	7.3	458 and 488	543 for DsRed	535	630 for DsRed	[46]
pHusion mRFP1-^AVNAS^-EGFP	5.8	488	558 or 585	500–550	600–630	[47]

a not determined/not applicable.

**Table 4.** Select examples of pH-sensitive FPs targeted to cytosol, nucleus, and endoplasmic reticulum.

**Sensor**	**Tag**	**pH**	**Organism/Cell Type**	**Instrument^a^**	**Reference**
Cytosol						

E^2^GFP		7.2–7.3	CHO, U-2 OS cells	Micro	[36]
E^2^GFP		7.3–7.5	Rat hippocampal neuron	Micro	[48]
deGFP4		7.4–7.7	Rat hippocampal neuron	Micro	[48]
GFP-F64L/S65T		7.4	CHO-1K cells	Micro	[11]
EYFP		7.4	HeLa cells	Micro	[12]
SypHer		7.2	HeLa cells	Micro	[41]
pHRed		7.4	Neuro 2A cells	Micro	[40]
pHERP		7.2–7.5	Vero, CFT1, CHO cells	Micro	[44]
ClopHensor		7.3, 7.7	WSS1, PC12, CHO cells	Micro	[45,46]
ClopHensor (H148G/V224L)		7.5–7.7	CHO cells	Micro	[46]
PalmPalm-ClopHensor	Palmitoylation site	7.3	WSS1, PC12 cells	Micro	[46]
GFpH	C-terminus α1B-adrenoreceptor	6.8	COS-7, CHO-K1 cells	Micro	[43]
Caveolin-E^1^GFP	Caveolin-1	7.3	HeLa cells	Micro	[50]
hCNT3-mNectarine	Cytosolic site of hCNT3	6.5–7.5	HEK cells	Micro	[25]
RaVC		7.4–7.7	*A. niger*	Micro	[33,34]
pHluorin		6.4–7.5^c^	*S. cerevisiae* FL100, S288c, ∑1278, ORY001, BY4742	Spec	[52–54]
pHluorin, pHluorin2		7.0–7.5^c^	*S. cerevisiae* H4307, H3909, BY4743	Micro Flow	[31,55]
pHluorin		7.2–7.3	*Schizo. pombe*	Micro	[49]
pHluorin		7.0^c^	*C. glabrata* BG14	Spec	[51]
pHusion		6.4	*Arabidopsis* mesophyll cell	Micro	[47]
pHGFP		6.5–7.0	*A. thaliana* root cap	Micro	[15]
pHGFP		7.0–7.3	*A. thaliana* meristem cells	Micro	[15]
PRpHluorin		7.3	*A. thaliana*	Micro	[4]

Nucleus						

NLS–PRpHluorin	PKKKRKV	7.2	*A. thaliana*	Micro	[4]
pHluorin-NLS	PKKKRKV	7.3	*Schizo. pombe*	Micro	[49]

Endoplasmic reticulum						

pHluorin-KDEL	KDEL at C-terminus of FP	b	Rat hippocampal neurons	Micro	[56]
GFP-F64L/S65T-SEKDEL	SEKDEL at C-terminus of FP	b	CHO-K1, LLC-PK1 cells	Micro	[11]
PpHluorin–HDEL	HDEL at C-terminus of FP	7.1	*A. thaliana*	Micro	[4]

a Micro, FLIM, spec, flow stand for microscopy, fluorescence life time microscopy, spectrophotometry and flow cytometry, respectively;

b Only pH changes are presented in the paper;

c Dependent on carbon source, external pH.

**Table 5.** Select examples of pH-sensitive FPs targeted to Golgi.

	**Sensor**	**Tag**	**pH**	**Organism/Cell Type**	**Instrument^a^**	**Reference**
*Medial/ trans*	GT-EGFP, GT-ECFP, GT-EYFP	β-1, 4-galactosyltransferase (N-terminal 81aa)	6.6	HeLa CHO-K1, LLC-PK1 cells	Micro	[11,12]
*Medial/ trans*	GT-EGFP/ GT-ECFP	β-1, 4-galactosyltransferase	6.4	HeLa cells	Micro	[61]
*Medial/ trans*	GT-EYFP/ GT-ECFP	β-1, 4-galactosyltransferase	6.4	HeLa cells	Micro	[61]
*Medial/ trans*	GT-EGFP	β-1, 4-galactosyltransferase	6.8	Cancer cells MCF-7, HT-29, SW-48 cells	Micro	[57]
*Medial/ trans*	GT-EGFP	β-1, 4-galactosyltransferase	6.1–6.3	COS7, CaCo2 cells, fibroblasts	Micro	[57]
*Medial/ trans*	ST-pHERP	2,6-sialyltransferase (1-70 aa)	6.4–6.6	CFT1, Vero, CHO, HeLa cells	Micro	[44]
TGN	TGN38-pHluorin	Trans Golgi network protein	6.4	HEK cells	Micro	[59]
TGN	PpHluorin-BP80 (Y612A)	BP-80 vacuolar sorting receptor	6.3	*A. thaliana*	Micro	[4]
*Cis*	ManI–PpHluorin	mannosidase	6.8	*A. thaliana*	Micro	[4]

a Micro, FLIM, spec, flow stand for microscopy, fluorescence life time microscopy, spectrophotometry and flow cytometry.

**Table 6.** Select examples of pH-sensitive FPs targeted to subcellular compartments of secretory and endocytic pathway.

	**Sensor**	**Tag**	**pH**	**Organism/Cell Type**	**Instrument^a^**	**Reference**
Perforin containing granules	DsRed-FasL-pHluorin	Fas ligand	b	NKL cells	Micro	[62]
BDNF containing vesicles and Golgi	SytIV-pHluorin	Synaptotagmin-IV	b	Rat hippocampal neurons	Micro	[63]
Synaptic vesicles	pHluorin-syt	Luminal domain of synaptotagmins	b	Rat hippocampal neurons	Micro	[67]
Synaptic vesicles	syp-pHTomato	Synaptophysin	b	HEK, rat hippocampal neurons	Micro	[24]
Synaptic vesicles	VAMP2-pHTomato	Synaptobrevin/ VAMP2	b	HEK, rat hippocampal neurons	Micro	[24]
Synaptic vesicles	GFP-F64L/S65T-	Synaptobrevin/ VAMP2	b	CHO-K1, LLC-PK1 cells	Micro	[11]
Synaptic vesicles	VAMP-EGFP	Synaptobrevin/ VAMP2	b	Rat hippocampal neurons	Micro	[14]
Synaptic vesicles	synapto-e_s_-pHluorin	Synaptobrevin/ VAMP2	b	Rat hippocampal neurons	Micro	[14]
Synaptic vesicles	VGLUT1-2xmOrange2	Glutamate transporter 1	b	Rat hippocampal neurons	Micro	[23]
Synaptic vesicles	vGluTpH	Glutamate transporter 1	b	Rat hippocampal neurons	Micro	[24]
Synaptic vesicles	vGluT-pHl	Glutamate transporter 1	b	Mouse hippocampal neurons	Micro	[66]
Synaptic vesicles	vGluT-pHl	Glutamate transporter 1	b	Mouse hippocampal neurons	Micro	[66]
Synaptic vesicles	syp-pHl	Synaptophysin 1	b	Mouse hippocampal neurons	Micro	[66]
PTH1R containing vesicles	hPTH1R-pHluorin2	human parathyroid hormone 1 receptor	b	HEK293 cells	Micro	[30]
Secretory garnules	CgA-ECFP	Chromogranin B	5.5	PC12 cells	FLIM	[69]
Vesicles/ endoplasmic reticulum	pHluron-GluR-D	signal peptide-pHluorin-glutamate receptor	b	Rat hippocampal neurons	Micro	[56]
Insulin secretory granules	Phogrin-pHluorin	Signal peptide-pHluorin-phogrin	b	MIN6 cells	Micro	[68]
Insulin secretory granules	Insulin-pHluorin	Prepro-insulin	b	MIN6 cells	Micro	[68]
Large dense-core vesicles LDCV	NPY-ClopHensor	Neuropeptide Y, N-terminal signal	5.2, 5.6	PC12, WSS-1 cells	Micro	[45]
Early endosomes	Cellubrevin-r-pHluorin	Cellubrevin	5.9	HEK cells	Micro	[59]
Endosomes	Tat-E^1^GFP	Transactivator protein of human immunodeficiency virus-1	6.8 early/ 5.8–6.5	HeLa cells	Micro	[50]
Multivesicular bodies	PRpHluorin–AtVSR2	Vacuolar-sorting receptor 2	6.2	*A. thaliana*	Micro	[4]
Vacuoles	aleurain–PRpHluorin	Aleurain	5.2	*A. thaliana*	Micro	[4]
Vacuoles	RaVC		6.2–6.5	*A. niger*	Micro	[33]

a Micro, FLIM, spec, flow stand for microscopy, fluorescence life time microscopy, spectrophotometry and flow cytometry;

b Only pH changes are presented in the paper.

**Table 7.** Select examples of pH-sensitive FPs targeted to peroxisomes, mitochondria, and other specialized compartments.

	**Sensor**	**Tag**	**pH**	**Organism/Cell Type**	**Instrument^a^**	**Reference**
Peroxisomes						

	pHluorin-SKL	SKL at C-terminus	6.9–7.1	CHO cells, human foreskin fibroblasts	Micro	[70]
	pHluorin-SKL	SKL at C-terminus	8.0	*S. cerevisiae* BY4743	Flow	[55]
	PRpHluorin-SRL	SRL at C-terminus	8.4	*A. thaliana*	Micro	[4]

Other specialized compartments						

Cell cortex	pHMA	moesin 140 aa C-terminus actin binding domain	7.0–7.4	*Drosophila* S2 cells	Micro	[71]
Apoplasts	Apo-pHusion	Chitinase	b	*A. thaliana* mesophyll cell	Micro	[47]
Plastid stroma/ chloroplasts	RecA–PRpHluorin	Rubisco activase (N-terminus)	7.2	*A. thaliana*	Micro	[4]

Mitochondria								

	mtAlpHi	COX-IV (1-12aa)	8.1	HeLa cells	Micro	[20]
	MitoSypHer	COX-VIII (1-25aa)	7.6	HeLa cells	Micro	[39]
	ECFPmito, EYFPmito	COX-IV (1-12aa)	8.0/7.9	HeLa/ cardiomyocytes	Micro	[12]
	mito-pHluorin	COX-IV (1-12 aa)-RSGI _linker_	7.7	CV-1 cells	Micro	[72]
	COX8-GFP-F64L/S65T	COX-VIII (1-25aa)	>7.5	CHO-K1, LLC-PK1	Micro	[11]
	COX8-pHRed	COX-VIII (1-25aa)	8.0	Neuro 2A cells	Micro	[40]
	COX4-pHluorin	COX-IV (1-25aa)	7.7	*S. cerevisiae* BY4743	Flow	[55]
	Mito-PpHluorin	β-subunit F1-ATPase	8.1	*A. thaliana*	Micro	[4]

a Micro, FLIM, spec, flow stand for microscopy, fluorescence life time microscopy, spectrophotometry and flow cytometry;

b Only pH changes are presented in the paper.
